# Single Amino Acid Substitution in the Matrix Protein of Rabies Virus Is Associated with Neurovirulence in Mice

**DOI:** 10.3390/v16050699

**Published:** 2024-04-28

**Authors:** Michiko Harada, Aya Matsuu, Yoshihiro Kaku, Akiko Okutani, Yusuke Inoue, Guillermo Posadas-Herrera, Satoshi Inoue, Ken Maeda

**Affiliations:** 1 Department of Veterinary Science, National Institute of Infectious Diseases, 1-23-1 Toyama, Tokyo 162-8640, Japan; hmichiko@niid.go.jp (M.H.); matsuu@niid.go.jp (A.M.); ykaku@niid.go.jp (Y.K.); okutani@niid.go.jp (A.O.); yinoue@niid.go.jp (Y.I.); posadasg@ims.u-tokyo.ac.jp (G.P.-H.); sinoue@niid.go.jp (S.I.); 2 Joint Graduate School of Veterinary Medicine, Yamaguchi University, 1677-1 Yoshida, Yamaguchi 753-8515, Japan

**Keywords:** rabies virus, mouse neuroblastoma cell, matrix protein, amino acid substitution

## Abstract

Rabies is a fatal encephalitic infectious disease caused by the rabies virus (RABV). RABV is highly neurotropic and replicates in neuronal cell lines *in vitro*. The RABV fixed strain, HEP-Flury, was produced via passaging in primary chicken embryonic fibroblast cells. HEP-Flury showed rapid adaptation when propagated in mouse neuroblastoma (MNA) cells. In this study, we compared the growth of our previously constructed recombinant HEP (rHEP) strain—based on the sequence of the HEP (HEP-Flury) strain—with that of the original HEP strain. The original HEP strain exhibited higher titer than rHEP and a single substitution at position 80 in the matrix (M) protein M(D80N) after incubation in MNA cells, which was absent in rHEP. *In vivo*, intracerebral inoculation of the rHEP-M(D80N) strain with this substitution resulted in enhanced viral growth in the mouse brain and a significant loss of body weight in the adult mice. The number of viral antigen-positive cells in the brains of adult mice inoculated with the rHEP-M(D80N) strain was significantly higher than that with the rHEP strain at 5 days post-inoculation. Our findings demonstrate that a single amino acid substitution in the M protein M(D80N) is associated with neurovirulence in mice owing to adaptation to mouse neuronal cells.

## 1. Introduction

Rabies is a lethal zoonotic disease, which causes encephalitis in almost all mammalian species [1]. An estimated 59,000 rabies cases are reported worldwide annually [2], and over 99% of human rabies cases are transmitted from dogs [3]. The World Health Organization (WHO) is promoting “the global strategic plan to end human deaths from dog-mediated rabies by 2030: Zero by 30” [4,5].

Rabies is caused by rabies virus (RABV) infection. RABV is a non-segmented, single negative-stranded RNA virus belonging to the *Lyssavirus* genus of the *Rhabdoviridae* family [1,3]. The viral genome encodes six structural proteins: the nucleoprotein (N protein), phosphoprotein (P protein), matrix (M) protein, glycoprotein (G protein), and large RNA-dependent RNA polymerase (L protein) [3,6]. After infection, RABV shows high neurotropism [7,8], resulting in its migration from the peripheral nervous system to the central nervous system [9,10]. RABV can easily replicate in neurological cell lines, such as mouse neuroblastoma (MNA) cells [11,12], mouse neuroblastoma (N2a) cells [13], and human neuroblastoma (SK-N-SH) cells [14]. To produce attenuated strains, street strains isolated from animals have been serially passaged into neuronal cells [15], baby hamster kidney fibroblasts (BHK cells) [16,17], and suckling mice [18], showing multiple substitutions. Major changes occur in the G protein after passage, and it is associated with multiple functions, including pathogenicity, antigenicity, and cell entry [3,6,7,19,20]. Position 333 in the G protein is associated with pathogenicity and leads to changes in the mortality of infected mice [8,21,22]. The relationship of the M protein with pathogenicity and neurovirulence is not known in detail, but it has been reported that the position 95 in this protein affects viral pathogenicity in mice [23].

The Flury strain of RABV isolated from a girl who died of rabies was transferred to 1-day-old chicks in 1940 [24]. Subsequently, the Flury strain was passaged over 180 times in chicken eggs to generate the high-egg-passage Flury strain (HEP-Flury) [21,24,25,26]. HEP-Flury was propagated in primary chicken embryo fibroblast cells for use as a human rabies vaccine strain [24], and a chick embryo cell-adapted HEP-Flury small-plaque-forming (CEF-S) strain was produced [25]. After intracerebral inoculation, HEP-Flury was highly attenuated. It is lethal in suckling mice and not adult mice [8]. The HEP-Flury strain can replicate, yielding high titers in MNA cells similar to other RABV strains [7,8]. 

In this study, our previously constructed recombinant HEP (rHEP) strain, based on the sequence of the HEP (HEP-Flury) strain from our laboratory, was characterized. We compared the propagation of the two strains in MNA cells. Infection with the original HEP strain produced significantly higher titers than rHEP strain despite the same sequence. Furthermore, we found a single substitution in the M protein between rHEP and the virus recovered from MNA cells infected with HEP. We characterized a single substitution in the M protein, M(D80N), and analyzed the virus propagation in MNA cells and its virulence in a mouse model. The results suggested that one substitution in the M protein plays a crucial role in the neuropathogenesis of RABV.

## 2. Materials and Methods

### 2.1. Cells and Viruses

MNA and BHK-21 cells were grown at 37 °C in Eagle’s minimum essential medium (MEM) (Nacalai Tesque, Kyoto, Japan) supplemented with 10% heat-inactivated fetal bovine serum (FBS) (Gibco, Grand Island, NY, USA). Chicken embryo fibroblast cells (DF-1) (CRL-12203; American Type Culture Collection (ATCC), Manassas, VA, USA) were maintained at 39 °C in Dulbecco’s modified Eagle’s minimum essential medium (DMEM) (Nacalai Tesque) supplemented with 10% heat-inactivated FBS.

The RABV used in this study was a laboratory strain of HEP-Flury (HEP) (Accession Number: LC785439), which was originally stocked in our laboratory after two rounds of propagation in MNA cells. Cloned HEP (cHEP) and cHEP-M(D80N) were cloned following the limiting dilution method using the supernatant of HEP-infected MNA cells at 2 d.p.i.

### 2.2. Reverse Transcription-Polymerase Chain Reaction (RT-PCR)

RABV RNA was extracted from the supernatant of infected cells using the QIAamp Viral RNA Mini Kit (QIAGEN, Hilden, Germany) according to the manufacturer’s protocol. To generate cDNA, reverse transcription was performed as previously described [27], and this cDNA was used for subsequent PCR amplification of RABV genome fragments. The cDNA templates were subjected to PCR using Prime STAR GXL (TaKaRa Bio, Shiga, Japan) and the respective primer sets (Supplementary Table S1). Purified PCR products were analyzed using the Sanger sequencing method. Sequence assembly and further analysis were conducted using GENETYX Ver.15 software (GENETYX, Tokyo, Japan) and Sequence Scanner (Thermo Fisher Scientific, Waltham, MA, USA).

### 2.3. Constructing and Rescuing Recombinant RABVs

To construct an infectious clone of RABV, PCR was performed using Prime STAR GXL (TaKaRa Bio), followed by Phusion Hot Start II High-Fidelity DNA Polymerase (Thermo Fisher Scientific) for the entire RABV genome. Mutant viruses were constructed by introducing point mutations through PCR using Prime STAR Max (TaKaRa Bio) and synthetic primers (Supplementary Table S1) with the indicated sequences. The assembled cDNA of the full RABV genome flanked by the hammerhead and delta virus ribozymes sequences (HamRz and HdvRz, respectively) was inserted between the *Kpn*I and *Pst*I sites of the pcDNA3.1 Zeo (+) plasmid (Thermo Fisher Scientific), as previously reported [28]. To construct helper plasmids, genes encoding N, P, G, and L proteins were amplified from HEP using conventional PCR (Supplementary Table S2) and cloned between the *Kpn*I and *Pst*I sites of the same vector. 

BHK-21 cells (3.0 × 10^5^ cells/well) were grown overnight in six-well plates and transfected with 1.2 µg of the full-length plasmid and 450 ng each of helper plasmids. Transfection was conducted using the TransIT-LT1 Transfection Reagent (Mirus Bio, Madison, WI, USA) following the manufacturer’s protocol. After five days, the supernatants were collected, and an aliquot was used for inoculation into MNA cells to confirm the presence of the virus using a direct fluorescent antibody test (DFAT). Supernatants from virus-positive wells (confirmed by DFAT) were propagated only once in MNA cells to obtain a virus stock. The nucleotide sequences of all rescued viruses were confirmed through sequencing. 

### 2.4. Virus Titration

MNA cells were seeded (4.0 × 10^4^ cells/well) in 96-well plates and incubated overnight at 37 °C with 5% CO_2_. Subsequently, serial 10-fold dilutions of the virus or the 10% brain emulsion in phosphate-buffered saline (PBS) were inoculated into the cells, which were then incubated at 37 °C for 2 days. The cells were fixed with 80% acetone for 30 min and stained with fluorescein isothiocyanate (FITC) anti-rabies monoclonal globulin (FUJIREBIO, Tokyo, Japan). Antigen-positive foci were counted under a fluorescence microscope (Nikon, Tokyo, Japan) and quantified in focus-forming units (FFU) per milliliter.

### 2.5. Comparing Viral Growth

MNA and DF-1 cells in 6-well plates were inoculated with each RABV strain at a multiplicity of infection (M.O.I.) of 0.05. After 1 h of adsorption, the cells were washed three times with MEM or DMEM, and 2 mL of MEM or DMEM supplemented with 10% heat-inactivated FBS was added to each well. The supernatant was collected at the indicated time points. Each experiment was independently repeated two or three times.

### 2.6. Animal Experiments

The animal study protocol was approved by the Committee for Animal Experimentation at our institute (NIID) (Approval Number: 123129). All possible efforts were made to minimize the suffering of laboratory animals. The mice were housed in the animal facility of the NIID. Six-week-old ICR (adult) mice (5/group) or suckling mice (10/group) (Japan SLC, Shizuoka, Japan) were inoculated intracerebrally with 10^5^ FFU/mouse of rHEP, rHEP-M(D80N), or MEM (as a negative control “mock”). The body weights of adult mice were monitored until 20 d.p.i., and mortality was recorded daily at 10 d.p.i. for suckling mice. The brains of the suckling mice were collected after death or euthanasia. Additionally, six-week-old ICR (adult) mice (3/group) were intracerebrally inoculated with 10^5^ FFU/mouse of the recombinant strains, and brain samples were collected at 5 and 7 d.p.i. for DFAT and virus titration. Brain samples from suckling mice (10/group) and adult mice (3/group) were applied with a toothpick to a 3-well microslide glass (Matsunami, Osaka, Japan), and each well was air-dried. Slides were fixed in 10% formalin supplemented with 0.4% TritonX-100 (Merck, Darmstadt, Germany) for 1 h and stained with FITC Anti-Rabies Monoclonal Globulin. The cells were counterstained with Evans Blue (FUJIFILM Wako Pure Chemical Corporation, Osaka, Japan).

### 2.7. Statistical Analysis 

Data are presented as mean ± standard deviation (S.D.). Statistical analyses were conducted using the two-way analysis of variance (ANOVA), followed by Tukey’s or Sidak’s multiple comparison tests. Unpaired *t*-tests were two-tailed. The log-rank test was used to analyze the Kaplan–Meier survival curves. Statistical analyses were performed using GraphPad Prism 9 (GraphPad Software, San Diego, CA, USA). Values of *p* < 0.05 were considered statistically significant.

## 3. Results

### 3.1. Comparison of Viral Growth between Original and Recombinant HEP

**Figure 1:**
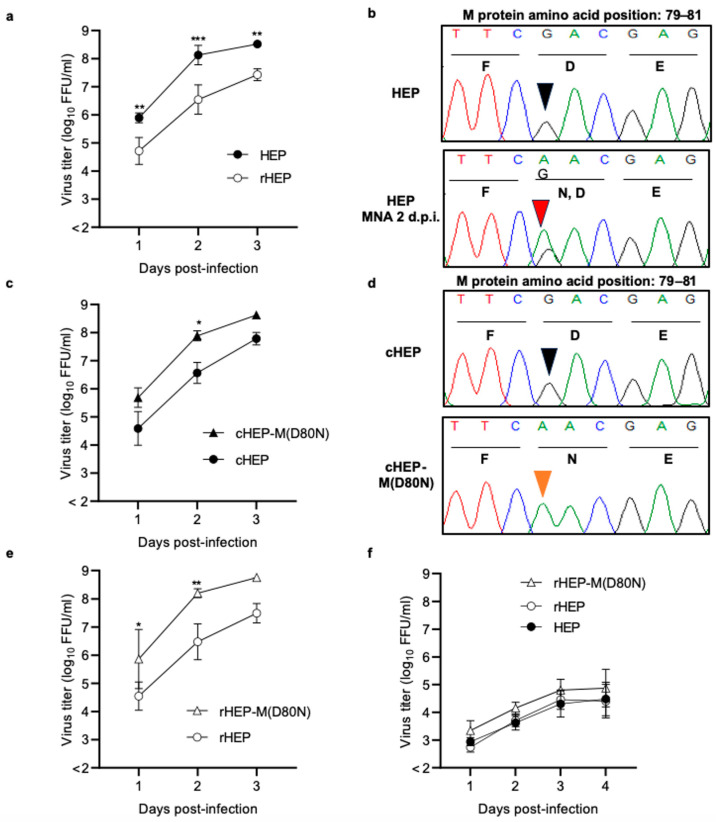
Comparison of viral growth and nucleotide sequences. Each strain was inoculated into MNA (**a**,**c**,**e**) or DF-1 cells (**f**) at a multiplicity of infection (M.O.I.) of 0.05. Growth curves of MNA cells were compared between HEP and recombinant HEP (rHEP) (**a**), cloned HEP (cHEP) and cHEP-M(D80N) (**c**), and rHEP and rHEP-M(D80N) (**e**). In DF-1 cells, the growth curves were compared with HEP, rHEP, and rHEP-M(D80N) (**f**). For viral titration, antigen-positive foci were counted under a fluorescence microscope and calculated as focus-forming units (FFU) per milliliter. The mean viral titer and standard deviation (S.D.) were calculated from two or three independent experiments. Significant differences are indicated (*: *p* < 0.05, **: *p* < 0.01, ***: *p* < 0.001) after two-way analysis of variance (ANOVA) followed by Tukey’s test. Nucleotide sequences from the original HEP strain and the supernatant of MNA cells infected with HEP at 2 days post-infection (d.p.i.) (**b**) and those from cHEP and cHEP-M(D80N) (**d**) were compared. The sequences of these strains were determined and compared using GENETYX Ver.15 (GENETYX, Tokyo, Japan) and a Sequence Scanner (Thermo Fisher Scientific, Waltham, MA, USA). The arrowhead points to nucleotide position 238 (amino acid position 80) in the M protein. Black, orange, and red arrowheads indicate guanine, adenine, and a mixture of adenine and guanine, respectively. HEP: high-egg-passage Flury laboratory strain.

Recombinant HEP (rHEP) was obtained via transfection of BHK-21 cells with the full-length plasmid and four helper plasmids encoding N, P, G, and L proteins. Supernatants were propagated only once in MNA cells to obtain a virus stock, and the nucleotide sequence of rHEP was confirmed to be identical to that of the original HEP (DDBJ Accession No. LC785439). A significant difference was observed between original HEP and rHEP when we compared their viral growth in MNA cells at an M.O.I. of 0.05 (Figure 1a), resulting in the original HEP growing significantly more in MNA cells at 1–3 d.p.i. than rHEP. We verified that the sequences of original HEP and rHEP were identical via Sanger sequencing, and then, we compared the nucleotide sequences of HEP collected from the supernatant at 2 d.p.i. The results revealed a single mixture of nucleotide sequences at position 238 (guanine and adenine) in the M gene of the HEP strain, resulting in a mixture of aspartic acid (D) and asparagine (N) at position 80 in the M protein (Figure 1b). In contrast, rHEP showed no change in amino acid content. Subsequently, we examined the sequences of HEP after serial passages in MNA cells, which resulted in an increase in the substitution from guanine to adenine at position 238 (Supplementary Figure S1). As HEP was highly passaged in chicken embryo cells, the viral growth of HEP and rHEP in DF-1 was compared. There were no significant differences in viral growth between HEP and rHEP (Figure 1f). Furthermore, there was no change in the HEP nucleotide sequence after propagation in DF-1 cells (Supplementary Figure S2).

### 3.2. Comparing Viral Growth of Limiting Dilution Viruses

After the propagation of HEP in MNA cells, the viral solution contained a mutant with a substitution of M(D80N). cHEP and cHEP-M(D80N) were cloned via limiting dilution from the supernatant collected at 2 d.p.i.. Nucleotide sequence analysis confirmed only one substitution among the cloned viruses (Figure 1d). cHEP and cHEP-M(D80N) strains were inoculated into MNA cells, and their viral growth was compared (Figure 1c). The cHEP-M(D80N) grew significantly better than cHEP.

### 3.3. Comparison of Viral Growth of Recombinant HEP-M(D80N) 

To confirm the enhancement in viral growth caused by the single substitution in the M protein, M(D80N), the recombinant HEP-M(D80N) strain, rHEP-M(D80N), was constructed using reverse genetics, and the viral growth of rHEP-M(D80N) was compared with that of rHEP. rHEP-M(D80N) grew significantly better in MNA cells than rHEP (Figure 1e) but not in DF-1 cells (Figure 1f). These results indicate that only one substitution of M(D80N) enhanced viral growth in MNA cells.

### 3.4. Pathogenicity of Recombinant HEP-M(D80N)

**Figure 2:**
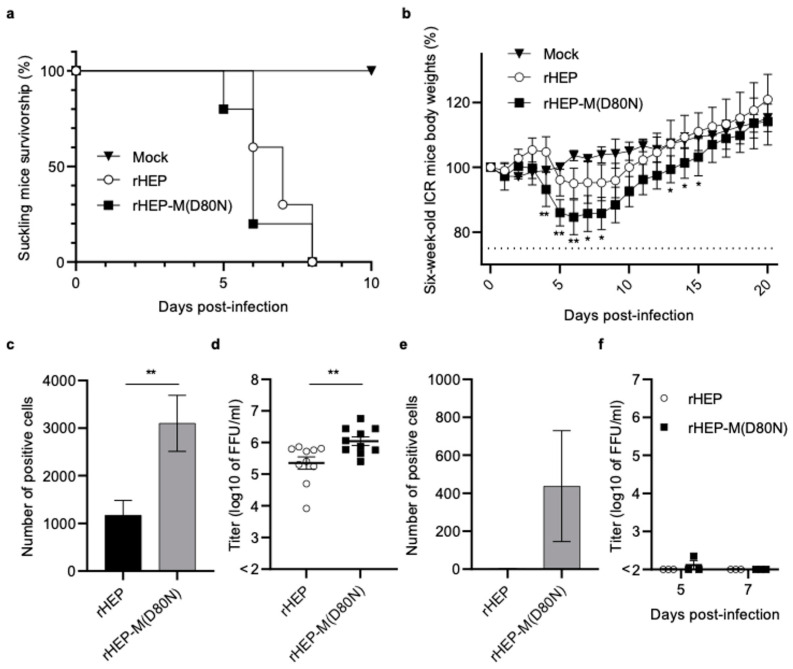
Comparison of pathogenicity between rHEP and rHEP-M(D80N) in suckling and 6-week-old mice. Suckling (*n* = 10/group) (**a**,**c**,**d**) and 6-week-old mice (*n* = 5/group) (**b**,**e**,**f**) were inoculated via intracerebral injection of 10^5^ FFU of the respective virus per mouse, or with an equivalent volume of medium (mock). Plots showing the survival rate of suckling mice using a Kaplan–Meier plot (**a**) and relative body weights (normalized to baseline) of 6-week-old mice (**b**). Body weight data are presented as the mean, and error bars represent the S.D. of each group. Significant differences are indicated (*: *p* < 0.05, **: *p* < 0.01) in the relative body weights between rHEP and rHEP-M(D80N) after the application of two-way ANOVA followed by Tukey’s test. The number of RABV-positive cells and viral titers in brain samples from all suckling mice (*n* = 10/group) after death (**c**,**d**) and adult mice (*n* = 3/group) at 5 d.p.i. (**e**) or 5 and 7 d.p.i. (**f**) were examined. The brain tissue was placed on a slide using toothpick (**c**,**e**), fixed in 10% formalin supplemented with 0.4% Triton X-100 solution, stained with fluorescein isothiocyanate (FITC)-conjugated anti-rabies monoclonal globulin (FUJIREBIO, Tokyo, Japan), and examined under a fluorescence microscope. Positive cells were quantified using ImageJ software (National Institutes of Health, Bethesda, MD, USA). Means and S.D. were calculated from two independent experiments, and significant differences are indicated (**: *p* < 0.01) after application of the unpaired *t*-test followed by two-tailed tests. For viral titration, a 10% brain emulsion with phosphate-buffered saline (PBS) was prepared (**d**,**f**).

To compare the pathogenicity of rHEP and rHEP-M(D80N), 10^5^ FFU of each virus was inoculated intracerebrally into suckling ICR mice (Figure 2a) and 6-week-old adult ICR mice (Figure 2b). Suckling mice infected with rHEP or rHEP-M(D80N) showed neurological signs at 4 d.p.i. and subsequently died at 5–8 d.p.i. (Figure 2a). For both strains, all infected adult mice survived until 20 d.p.i. and showed no clinical signs other than body weight loss (Figure 2b). These inoculated adult mice exhibited loss of body weight at 4 d.p.i and recovered from 7 d.p.i. Notably, mice inoculated with rHEP-M(D80N) showed a more significant body weight loss than those inoculated with rHEP.

A DFAT was performed using RABV nucleoprotein monoclonal antibody to compare viral antigens in the brain. The number of fluorescence-positive cells in the brains of suckling mice inoculated with rHEP-M(D80N) was significantly higher than that in those inoculated with rHEP (Figure 2c and Supplementary Figure S3). Additionally, the virus titer in mice inoculated with rHEP-M(D80N) was significantly higher than that with rHEP when the 10% brain emulsion was titrated (Figure 2d). In adult mice, the number of fluorescence-positive cells was higher in the brains inoculated with rHEP-M(D80N) than in those inoculated with rHEP at 5 d.p.i., during the start of body weight loss (Figure 2e and Supplementary Figure S4). The titer of the 10% brain emulsion was almost undetectable at 5 and 7 d.p.i. (Figure 2f), and no positive cells were found in brain samples from either group at 7 d.p.i. (Supplementary Figure S4).

## 4. Discussion

Our findings demonstrate that our laboratory strain (HEP) grew better in MNA cells than rHEP. The nucleotide sequences of HEP collected at 2 d.p.i. indicated a mixture of adenine and guanine in the original HEP at position 238 of the M gene. After the passage of HEP in MNA cells, the proportion of adenine increased, and there was little guanine in the third passage of HEP. This mutation induced a change in the amino acid from aspartic acid (D) to asparagine (N) at position 80 in the M protein. Subsequently, we cloned the viruses cHEP and cHEP-M(D80N) via limiting dilution and constructed recombinant HEP-M(D80N). Viruses with the M(D80N) substitution grew in MNA cells significantly better than those without the substitution, suggesting that this M(D80N) substitution enhanced viral growth in MNA cells. In contrast, there was no significant difference in viral growth in chicken DF-1 cells, regardless of the mutation. We hypothesize that the M(D80N) substitution may be important for viral growth in mouse neuronal cells.

Adult mice inoculated with rHEP-M(D80N) showed significantly reduced body weights compared to those inoculated with rHEP, and the number of RABV-positive cells in the rHEP-M(D80N)-infected mouse brains was significantly higher than that in the rHEP-infected mouse brains. Furthermore, the viral titers in the brains of rHEP-M(D80N)-infected suckling mice were significantly higher than those in the brains of rHEP-infected mice. These results indicate that mutation M(D80N) enhances neurovirulence by enhancing viral growth in the mouse brain. 

M protein is crucial in RABV replication and morphogenesis, including viral assembly and budding [20,29,30]. The late-budding domain of the M protein (amino acid position: 35–38) is related to replication and pathogenicity [31,32], and the intermediate filament protein (desmin) interacts with the M protein to regulate viral replication [33]. Additionally, the amino acid at position 95 of the M protein is associated with cell membrane disruption [23,34]. The RABV M protein is associated with stimulation of the JAK-STAT pathway through its interaction with the P protein and inhibition of the interferon-stimulated response element (ISRE) and IFN-α and IFN-β production [35], and the amino acids at positions 77, 100, 104, and 110 in the M protein were associated with this pathway [35,36], resulting in the enhancement in viral replication. Moreover, in RABV strains SN and SB, the exchange of the corresponding M proteins leads to changes in growth in mouse neuroblastoma cells [37]. These reports and our results demonstrate that the M protein is critical for viral replication in mouse neuroblastoma cells. 

There was no change in the nucleotide sequence of the M gene after the rHEP strain was passaged ten times in MNA cells. Viral sequences from the supernatant on day 4 after the inoculation of DF-1 cells with HEP did not show any change in the M gene (Supplementary Figure S2). These results suggest that the M(D80N) substitution is important for increased replication in mouse neuronal cells but is not necessary. The rHEP-M(D80N) strain replicated well in the mouse brain, and the number of RABV-positive cells in the brains of mice inoculated with rHEP-M(D80N) was higher at 5 d.p.i. Amino acid substitution M(D80N) is crucial in neurovirulence by enhancing viral growth in neuronal cells. Further studies are required to elucidate the underlying mechanisms. 

In conclusion, this novel finding demonstrates that the M protein is associated with neurovirulence in mice owing to adaptation to mouse neuronal cells. The amino acid substation—that is, M(D80N)—may play a crucial role in this adaptation.

## Data Availability

All data generated or analyzed during this study are included in this published article and its Supplementary Information files.

## Supplementary Materials

The following supporting information can be downloaded at: https://www.mdpi.com/article/10.3390/v16050699/s1, Figure S1: Comparison of nucleotide and amino acid sequences of original HEP-Flury after propagations in MNA cells; Figure S2: Nucleotide sequence of original HEP-Flury after propagation in chicken embryo fibroblast cells, DF-1; Figure S3: Direct fluorescent antibody test (DFAT) of brain samples of suckling mice inoculated with rHEP or rHEP-M(D80N); Figure S4: Direct fluorescent antibody test (DFAT) of brain samples of adult mice inoculated with rHEP or rHEP-M(D80N); Table S1: Primers used for PCR and construction of full genome for infectious clones; Table S2: Primers used for construction of helper plasmids.
