# Supplementary material for: Single Amino Acid Substitution in the Matrix Protein of Rabies Virus Is Associated with Neurovirulence in Mice
Source: Viruses. 2024 Apr 28;16(5):699. doi: 10.3390/v16050699 (PMC11125599; doi:10.3390/v16050699)
Supplement: Supplementary file 1 [file viruses-16-00699-s001.zip › viruses-2979607-supplementary.pdf]

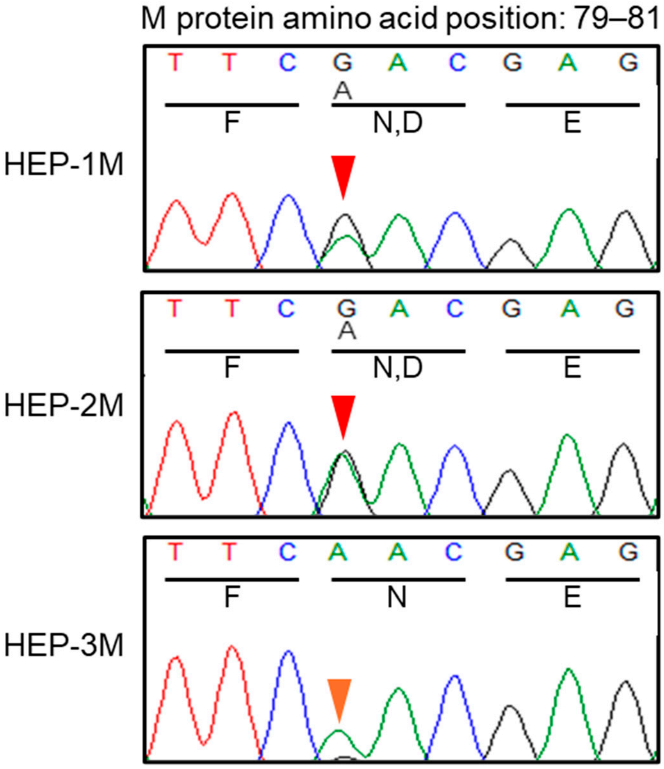

Supplemental Fig. S1 Comparison of nucleotide and amino acid sequences of original HEP-Flury after propagations in MNA cells.

The sequences of amino acids position 79 to 81 in the matrix (M) protein of propagated HEP strain after one, two, and three passages into MNA cells (HEP-1M, HEP-2M, and HEP-3M) are shown. Sequences of these strains were determined and compared using GENETYX Ver.15 (GENETYX, Tokyo, Japan) and a Sequence Scanner (Thermo Fisher Scientific, Waltham, MA, USA). At the nucleotide position of 238 (amino acid position 80) in the M protein, red arrowheads indicate a mixture of adenine and guanine, and the orange arrowhead indicates adenine.

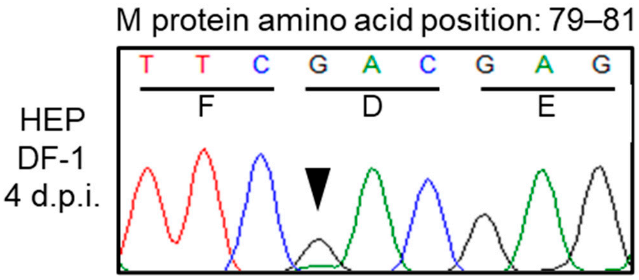

Supplemental Fig. S2 Nucleotide sequence of original HEP-Flury after propagation in chicken embryo fibroblast cells, DF-1.

The original HEP-Flury was inoculated to DF-1 cells at a multiplicity of infection (M.O.I.) of 0.05. The sequences were determined from the supernatant of DF-1 cells at 4 days post infection (d.p.i.) and compared using GENETYX Ver.15 (GENETYX, Tokyo, Japan) and a Sequence Scanner (Thermo Fisher Scientific, Waltham, MA, USA). The sequence at amino acid positions 79 to 81 in the matrix (M) protein are shown. Black arrowhead indicates guanine.

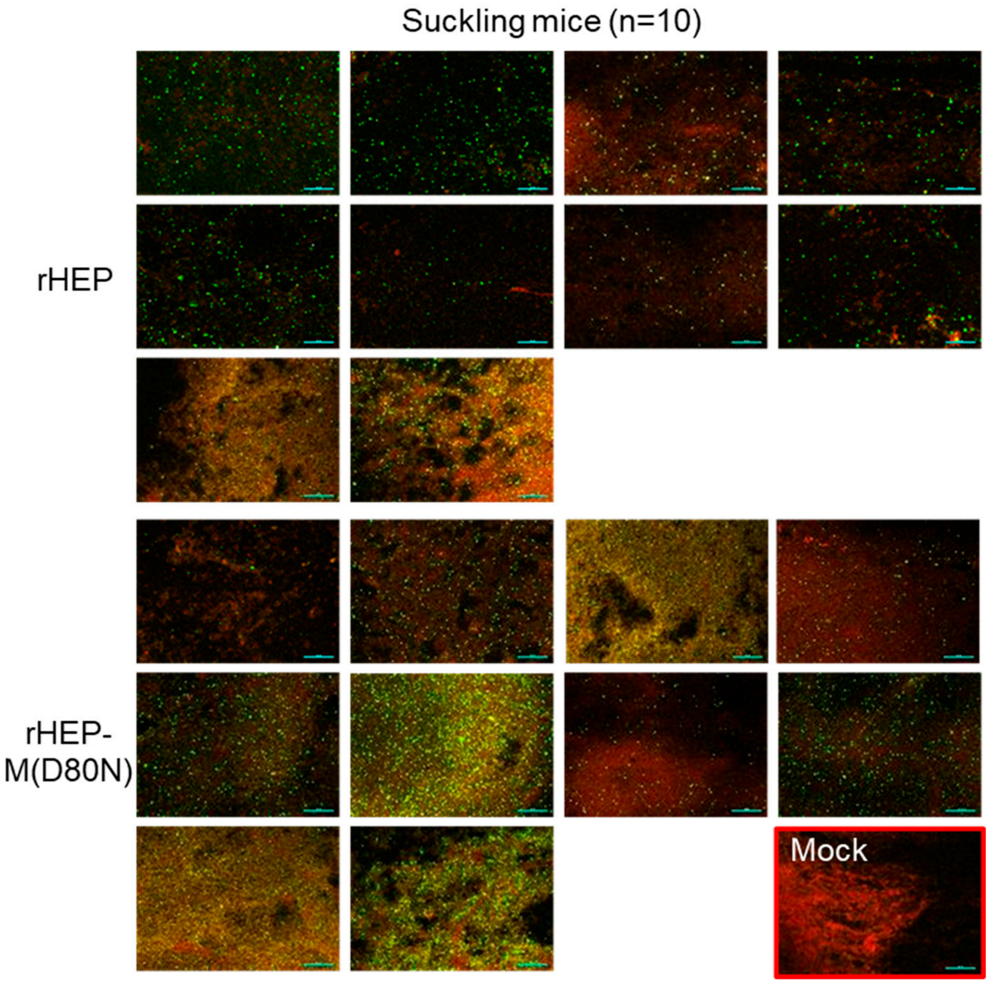

Supplemental Fig. S3 Direct fluorescent antibody test (DFAT) of brain samples of suckling mice inoculated with rHEP or rHEP-M(D80N).

Brain tissues were collected from suckling mice inoculated with either virus and applied to the slide with a toothpick. The slides were fixed in 10% formalin supplemented 0.4% Triton X-100 solution, stained with fluorescein isothiocyanate (FITC)-conjugated anti-rabies monoclonal globulin (FUJIREBIO, Tokyo, Japan), and examined under a fluorescence microscope. The stained samples were observed using NIS-Elements D version 5.20.00 imaging software (Nikon, Tokyo, Japan). RABV-positive cells appear green, while negative cells are stained red with Evans Blue. Scale bars, 100 μm; magnification, ×40. Brain samples from each of ten suckling mice that died at 5-8 d.p.i. after inoculation with either virus are shown.

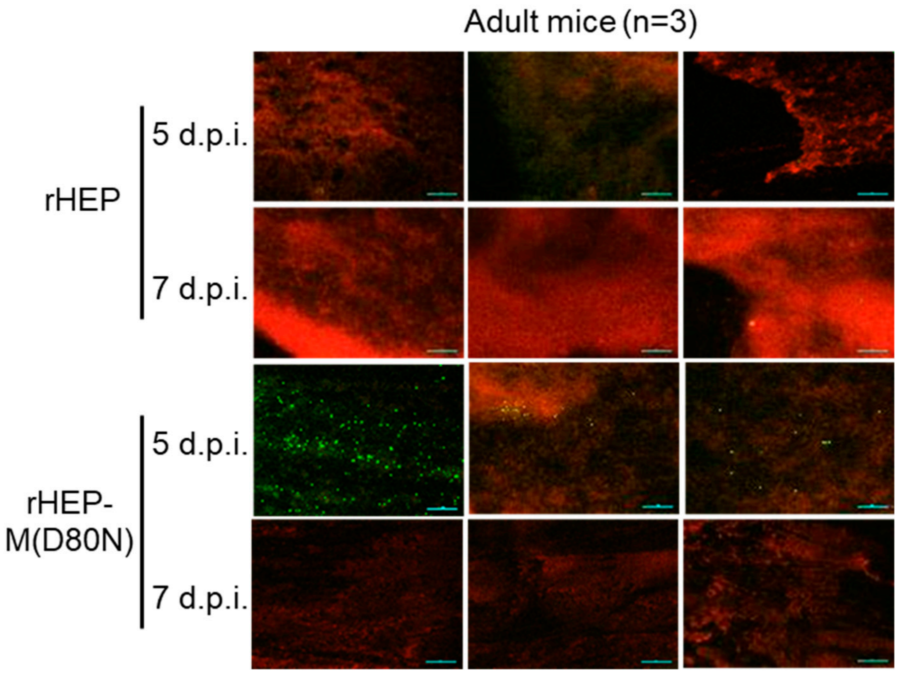

Supplemental Fig. S4 Direct fluorescent antibody test (DFAT) of brain samples of adult mice inoculated with rHEP or rHEP-M(D80N).

Brain tissues were collected from adult mice inoculated with either virus and applied to the slide using a toothpick. The slides were fixed in 10% formalin supplemented 0.4% Triton X-100 solution, stained with FITC-conjugated anti-rabies monoclonal globulin, and examined under a fluorescence microscope. The stained samples were observed using NIS-Elements D version 5.20.00 imaging software. RABV-positive cells appear green, while negative cells are stained red with Evans Blue. Scale bars, 100 μm; magnification, ×40. Brain samples from adult mice inoculated with either virus are shown. Samples were collected from three mice at 5 and 7 d.p.i.

Supplemental Table S1 Primers used for PCR and construction of the full genome of the infectious clones.

Primer name	Orientation	Sequence (5′→3′)	Position*
RABV 1	Forward	ACGCTTAACAACAAAACCAAAGAAG	1–25
	Reverse	TGAGCGATCTCAGCCTCYACTGATAG	2121–2096
RABV 2	Forward	CTTCCGTTCACTAGGCTTGAGTGGG	934–958
	Reverse	GGACCAAGTTTGTCTGGTATCG	3412–3391
RABV 3	Forward	CTATGGTCTGACATGTCTCTTCAG	3033–3056
	Reverse	GACTTGGAATAGAAATGGGCCAAGTC	5790–5765
RABV 4	Forward	TGTCCCCAACATCTTGAGGAACTC	5488–5511
	Reverse	CGCATTGGTGGATACTGTAGA	7912–7892
RABV 5	Forward	TACTAGCTCAAGGAGACAACCAGGT	7581–7605
	Reverse	AGCTGCATGGCGCACCTCTTGATC	10249–10226
RABV 6	Forward	CAGCTCAGGGGCTCTTATACTCAATC	9555–9580
	Reverse	ACGCTTAACAAATAAACAATAAAGAT	11925–11900
HEP-M_D80N	Forward	ATCATTCAACGAGATATACTCTGGGAA	2726-2752
	Reverse	ATCTCGTTGAATGATCTCAGAATATGC	2740-2714
Kpn_HamRz_HEP	Forward	ATAGGTACCTGTTAAGCGTCTGATGAGTCCGTGAGGACGAAACTATAGGAAAG<br>GAATTCCTATAGTCACGCTTAACAACAAAACCAAAGAAGAAGCA*	1–30
Pst_HdvRz_HEP	Reverse	CGGCTGCAGCGCCCTCCCTTAGCCATCCGAGTGGACGTGCGTCCTCCTTCGGA<br>TGCCCAGGTCGGACCGCGAGGAGGTGGAGATGCCATGCCGACCCACGCTTAA<br>CAAATAAACAATA*	11925–11905

Ribozyme sequences are underlined.

* The positions of the primers for PCR and plasmid construction were defined according to the genomic sequence of the HEP strain.

Supplemental Table S2 Primers used to construct helper plasmids

Primer name	Orientation	Sequence (5′→3′)	Position*
N protein	Forward	ATAGGTACCATGGATGCCGACAAG	67–85
	Reverse	CGGCTGCAGTTATGAGTCACTCG	1423–1410
P protein	Forward	ATAGGTACCATGAGCAAGATCTTTG	1511–1529
	Reverse	CGGCTGCAGTTAGCATGATGTGTAG	2408–2392
G protein	Forward	ATAGGTACCATGGTTCCTCAGGTTC	3318–3333
	Reverse	CGGCTGCAGTCACAGTCTGGTCTCG	4892–4877
L protein	Forward	ATAGGTACCATGCTGGATCCGGGA	5411–5425
	Reverse	CGGCTGCAGTTACAAACAACTGTAG	11794–11779

* The positions of the primers for PCR and plasmid construction were defined according to the genomic sequence of the HEP strain.
